# The steady-state response of the cerebral cortex to the beat of music reflects both the comprehension of music and attention

**DOI:** 10.3389/fnhum.2015.00436

**Published:** 2015-08-06

**Authors:** Benjamin Meltzer, Chagit S. Reichenbach, Chananel Braiman, Nicholas D. Schiff, A. J. Hudspeth, Tobias Reichenbach

**Affiliations:** ^1^Department of Bioengineering, Imperial College London, LondonUK; ^2^Tri-Institutional Training Program in Computational Biology and Medicine, Weill Cornell Medical College, New York, NYUSA; ^3^Howard Hughes Medical Institute and Laboratory of Sensory Neuroscience, The Rockefeller University, New York, NYUSA; ^4^Department of Neuroscience, Brain, and Mind Research Institute, Weill Cornell Medical College, New York, NYUSA

**Keywords:** auditory neuroscience, auditory cognition, auditory cortex, music processing, cortical response, cortical oscillations, auditory attention

## Abstract

The brain’s analyses of speech and music share a range of neural resources and mechanisms. Music displays a temporal structure of complexity similar to that of speech, unfolds over comparable timescales, and elicits cognitive demands in tasks involving comprehension and attention. During speech processing, synchronized neural activity of the cerebral cortex in the delta and theta frequency bands tracks the envelope of a speech signal, and this neural activity is modulated by high-level cortical functions such as speech comprehension and attention. It remains unclear, however, whether the cortex also responds to the natural rhythmic structure of music and how the response, if present, is influenced by higher cognitive processes. Here we employ electroencephalography to show that the cortex responds to the beat of music and that this steady-state response reflects musical comprehension and attention. We show that the cortical response to the beat is weaker when subjects listen to a familiar tune than when they listen to an unfamiliar, non-sensical musical piece. Furthermore, we show that in a task of intermodal attention there is a larger neural response at the beat frequency when subjects attend to a musical stimulus than when they ignore the auditory signal and instead focus on a visual one. Our findings may be applied in clinical assessments of auditory processing and music cognition as well as in the construction of auditory brain-machine interfaces.

## Introduction

Speech and music are fundamental forms of human communication (Juslin and Laukka, 2003). Humans possess the unique ability to analyze and comprehend speech and music with a remarkable degree of sophistication: we can understand a particular speaker even in a noisy environment with many other simultaneous conversations, and trained musicians can focus on a single instrument in an orchestral performance. The human ability to parse such complex acoustic scenes greatly exceeds that of current technology: speech-recognition systems, for instance, do well when following a single voice in a quiet environment but perform much worse when background noise is present.

The analyses of speech and music in the brain have recently been shown to share important neural resources (Patel, 2003, 2007; Koelsch et al., 2004; Koelsch, 2005; Sturm et al., 2014). Studies using magnetoencephalography (MEG) and functional magnetic resonance imaging (fMRI) found that unexpected and irregular chords activate Broca’s area as well as posterior temporal regions of the cortex (Maess et al., 2001; Koelsch et al., 2002, 2005; Tillmann et al., 2003). Investigations through fMRI as well as positron emission tomography (PET) have further identified cortical areas such as the superior and the transverse temporal gyri that are involved in the comprehension of music (Parsons, 2001; Morrison et al., 2003). These cortical areas are also crucially involved in the processing of language (Friederici, 2002; Bear et al., 2007). The underlying neural mechanisms through which the brain processes speech and music and by means of which it can selectively attend to a certain auditory signal despite background noise, however, remain largely unknown. A better understanding of these mechanisms could inspire technology for auditory-signal processing and could provide insight into the causes and potential treatments of auditory-processing disorders. Many elderly people, for instance, have difficulty with understanding speech in the presence of noise (Gordon-Salant and Fitzgibbons, 1993). This problem often results from impairment in the neural pathways that are responsible for auditory processing. Furthermore, 4% of people suffer from congenital amusia, a brain disorder in the processing of music (Ayotte et al., 2002; Henry and Mcauley, 2010). Amusia can also be acquired through brain damage. The specifics of such impairments, however, require further research.

Studies on the neural mechanism of auditory processing often employ simple acoustical signals. Important examples include amplitude-modulated pure tones, for which it has been shown that the neural activity of the cerebral cortex responds at the frequency of amplitude modulation (Hall, 2007). Whether attention to the auditory signal influences this auditory steady-state response (ASSR) remains debated: the magnitude of the response has been found to be modulated by attention in some studies (Ross et al., 2004; Skosnik et al., 2007; Müller et al., 2009) although other investigations have failed to detect such an effect (Linden et al., 1987; Skosnik et al., 2007; Saupe et al., 2009; Deng and Srinivasan, 2010). Electroencephalographic (EEG) experiments on intermodal attention, however, have revealed that attention to a frequency-tagged auditory stimulus as compared to attention to a visual stimulus may enhance the cortical response to the amplitude-modulated auditory input (Saupe et al., 2009; de Jong et al., 2010; Gander et al., 2010).

Recent studies have begun to investigate the ASSR in connection with more complex, naturalistic auditory signals. Specifically, an EEG experiment found that amplitude-modulated speech elicits an ASSR, and that the strength of the signal in the left temporal region of the cortex was stronger for unintelligible reversed speech than for intelligible forward speech (Deng and Srinivasan, 2010). Another recent study used MEG to show that amplitude-modulated music elicits an ASSR as well (Lamminmäki et al., 2014). It remains unclear, however, whether this response is modulated by the comprehension of music or by attention, and whether it can be measured by EEG, a more clinically applicable measurement technique. Moreover, the participants of the study found that amplitude modulation disrupted the quality of the music (Lamminmäki et al., 2014).

Other authors have used the event-related potential (ERP), that is, the brain’s response to a brief stimulus, to investigate auditory processing. The mismatch negativity, for instance, is a component of the ERP that occurs in response to a deviant stimulus in a sequence of otherwise similar stimuli (Hall, 2007; Näätänen et al., 2007). Recent studies found that the mismatch negativity is enhanced when a deviant sounds occurs among a sequence of familiar sounds rather than during a sequence of unfamiliar time-reversed sounds and that it is stronger for a deviant familiar sound then for a deviant unfamiliar sound when both occur among the same frequently presented stimuli (Jacobsen et al., 2005; Beauchemin et al., 2006). The mismatch negativity may be modulated by the familiarity of music as well: deviant stimuli consisting of familiar pitch changes elicit a greater response than stimuli with unfamiliar pitch changes (Brattico et al., 2001). It remains unclear, however, how more complex features such as comprehension of a whole musical tune or attention to it can be decoded from the listening brain.

Here we investigate the brain’s steady-state response to naturalistic music and how the neural response can represent music comprehension and attention to music. We inquire specifically whether non-invasive, clinically applicable EEG recordings of the neural response to continuous music allow assessments of subjects’ comprehension of a musical piece and of their attention to the music. Quantification of musical cognition from single-trial EEG measurements could lead to an objective assessment of amusia. It could also inspire brain-computer interfaces that employ a subject’s attention to communicate a choice or command. Because such clinical applications are likely to employ wearable EEG systems with few channels, we also explored how reliable information on music cognition could be obtained from only a few electrodes (Bonato, 2003; Casson et al., 2008).

Recent studies have demonstrated that delta- and theta-band cortical oscillations, which occur at respectively 0.5–4 and 4-7 Hz, provide information regarding speech processing. The rate of syllables and words in speech lies in the delta and theta frequency ranges, and neural oscillations in those frequency bands entrain to the envelope of a speech signal to which a person attends (Ding and Simon, 2012; Power et al., 2012; Horton et al., 2013; Peelle et al., 2013; Zion Golumbic et al., 2013). The neural entrainment to the envelope is stronger for an attended speech stream then for an unattended one (Horton et al., 2013) and may be stronger for an intelligible than for an unintelligible speech signal (Peelle et al., 2013; Ding and Simon, 2014; Ding et al., 2014). We investigated whether the cortical response to the natural rhythm of music analogously signals attention to and comprehension of music. Because the beat and meter of music typically lie in the frequency range of 1-7 Hz (Krumhansl, 2000; Justus and Bharucha, 2002; Large, 2008), we focused on the cortical response to rhythmic structure in the delta and theta frequency bands. Recent studies have shown that delta- and theta-band cortical oscillations synchronize to the beat and meter of simplified rhythmic stimuli (Nozaradan et al., 2011, 2012; Cong et al., 2013). This neural response is a promising candidate for reflecting the cognition of music.

Because the beat frequency is prominent in the envelope of a musical signal, the neural response to it can be viewed as an ASSR. This response is elicited by the beat of naturalistic music itself, so the perception of the naturalistic music that we employ here is not distorted by a superimposed amplitude modulation. This type of stimulus is accordingly well suited for studying the brain’s processing of music.

## Materials and Methods

### Participants

Eight young adult volunteers with normal hearing, of ages 20–30, right-handed, with no history of hearing or neurological problems, and without professional musical training, participated in the experiments. Six of the subjects were male and two female. Each participant underwent two 30-min EEG recordings on separate days that were typically spread over 2 weeks. All experimental methods were approved by the Imperial College Research Ethics Committee and all participants gave written informed consent before the experiment.

### Musical Pieces

**FIGURE 1:**
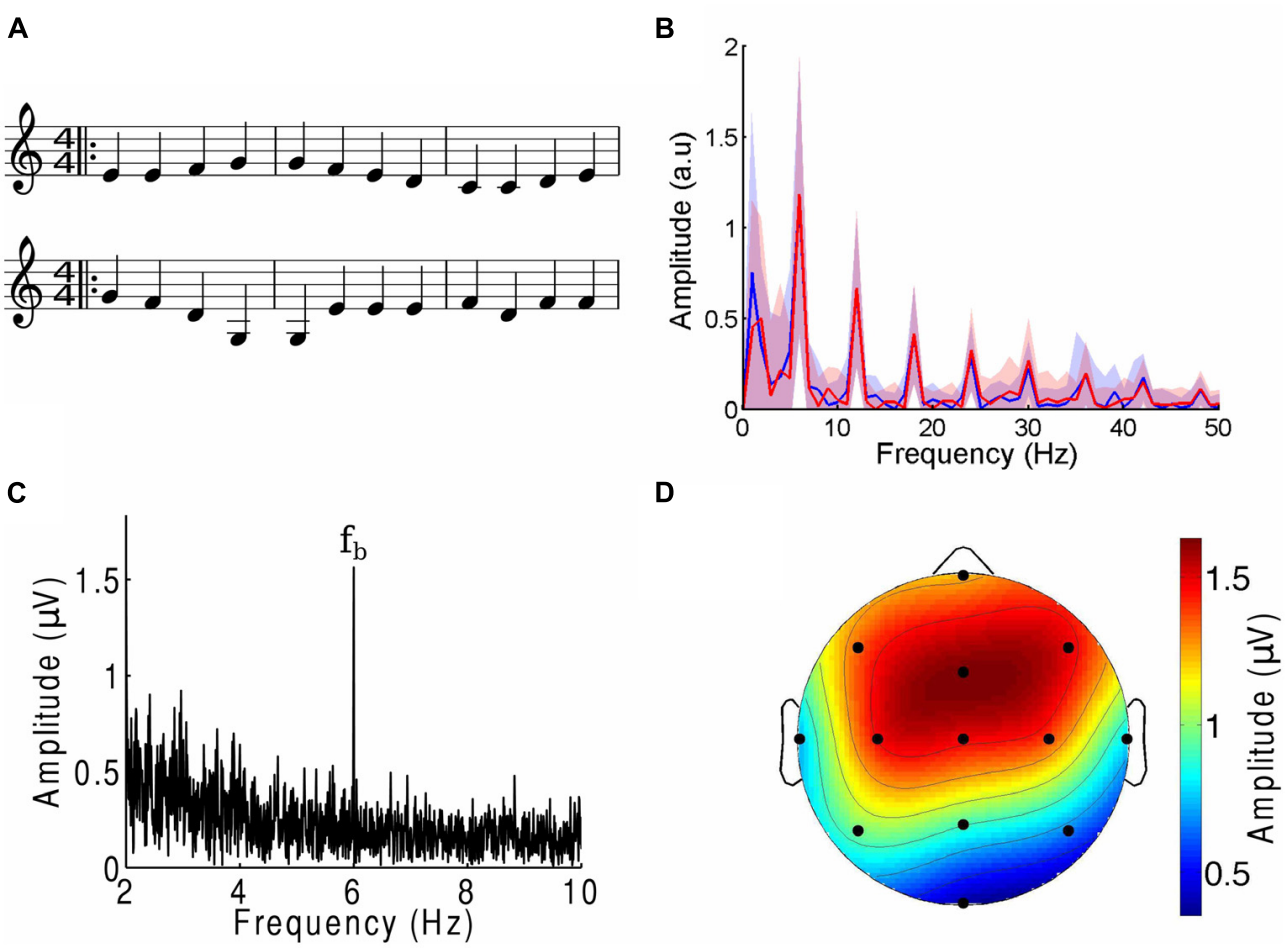
**The cortical response to the beat of music. (A)** Three bars from the musical score of a musical piece (OTJ, top) may be contrasted with its scrambled version (bottom). Note that the randomization occurs over the whole length of the musical piece, and not only over the three bars that are shown here. The notes in the first three bars of the musical piece are thus not all identical to those in the first three bars of the scrambled version. **(B)** The amplitude spectra of the envelope of a musical piece (FE, blue) and its scrambled version (red) show the same magnitudes at the beat frequency and its higher harmonics. Deviations between the two spectra are well below the noise (shaded areas denote the SEM). **(C)** The amplitude spectrum from a frontal channel in a representative subject in response to an attended musical piece contained a large response at the beat frequency *f*_b_ (6 Hz). **(D)** The response at the beat frequency was largest in the frontal area and smallest at the occipital pole. The scalp topographic map displays the amplitude of the electroencephalography (EEG) responses at the beat frequency in response to an attended musical piece, averaged over all subjects and trials.

Using the software Sibelius (Avid Technology, USA), we generated four melodies adapted from the main musical themes of *Für Elise* (Ludwig van Beethoven), *Eine Kleine Nachtmusik* (Wolfgang Amadeus Mozart), *Ode To Joy* (Ludwig van Beethoven, excerpt from the Ninth Symphony) and *Twinkle Twinkle Little Star* (popular English lullaby). They are referenced by the acronyms FE, EKN, OTJ, and TT, respectively. Each piece was edited to contain only a single melodic line without any accompaniment. The beat frequency was set to 6 Hz. The pieces were further manipulated such that one note occurred at every beat (**Figure 1A**). All pieces had notes centered within the same octave range and lasted two minutes. Despite the editing, the four musical tunes remained highly recognizable.

We then created scrambled versions of each musical piece by dividing the musical score into segments that ranged randomly from two to six notes in length, then randomly rearranging these sections (**Figure 1A**). Randomization was implemented through a custom-written MATLAB program that operated on an XML file generated in Sibelius. We verified that the randomization process retained the low-level structure of the tunes through two different approaches. The first approach, and one which is important in comparing the brain’s response to the beat of music, consisted of verifying that the amplitude spectra of the envelope of each musical piece and its randomized version were identical (**Figure 1B**). To this end we computed the envelope of the different musical pieces through a Hilbert transform. The amplitude spectrum and associated uncertainty were computed by dividing each two-minute envelope into 120 one-second segments, computing the frequency spectrum for every segment, and therefrom calculating the mean and SEM. We verified that the deviations between the amplitude spectra of the envelope of the musical tunes and their randomized versions were small and within the noise. Second, we investigated the frequency interval between two successive notes, divided by the frequency of the lower tone (pitch change). The mean of the relative pitch change between two neighboring tones was between 0.15 and 0.22 with a SD between 0.02 and 0.04. There was no statistically significant difference in the pitch changes between a musical tune and its scrambled versions (*p*-values of two-tailed Student’s *t*-tests were 0.13 or higher). Despite these statistical similarities, subjects confirmed that the scrambled musical pieces were non-sensical and unrecognizable. Examples of an excerpt from a musical piece and its scrambled version are shown in **Figure 1A**.

### Experimental Design

In the first experiment we tested differences in the neural response to the musical pieces and their scrambled counterparts. Subjects listened to the musical pieces as well as their scrambled versions while visually fixating at a stationary position. The experiment was divided into four trials. In each trial two EEG recordings, each two minutes in duration, were acquired. One recording was made during the presentation of a musical piece and the other recording during the presentation of the tune’s corresponding scrambled version. Whether the musical piece or the scrambled version was presented first was chosen at random. Each of the four trials employed a different musical piece and is referred to by the name of this piece in the following. The order of the presentation of the pieces was randomized between subjects. Subjects were asked whether they recognized the tunes to ensure that they were familiar with the melodies.

In the second experiment we tested attention-mediated effects on the cortical response to music. Subjects were presented with music streams and a printed excerpt from a novel. Again we performed four trials for every subject. During every trial subjects listened to a different musical piece, and subsequently the trial was referred to by the name of that piece. We randomized the order of the presentation of the musical pieces. In each trial, two EEG recordings lasting two minutes apiece were acquired. During one recording a subject was asked to attend the music and ignore the text, whereas for the other presentation the subject was instructed to read the text and disregard the music. Such an approach has been employed previously to identify an effect of attention on the auditory response to short tones (Tiitinen et al., 1993). The two tasks were presented in a random order. Different texts were used in the different trials to ensure maximal attention. Attention to the musical piece was verified through inquiring whether subjects recognized the tune, and attention to the text was verified through comprehension questions. All subjects answered these questions satisfactorily.

### EEG Recordings

We used scalp electrodes to measure the cortical responses of volunteers to music. To ensure attention and avoid distraction, each subject sat in a comfortable chair in a quiet room and was asked to keep his or her eyes open and fixated on an object straight ahead. Monopolar EEG signals were acquired using a biosignal amplifier (g.BSamp), active electrodes (g.LADYbird), a passive ground electrode (g.LADYbirdGND), and an Ag/AgCl active earclip electrode (g.GAMMAearclip; Guger Technologies, Austria). The analog voltage signals were band-pass filtered between 0.1 and 100 Hz and amplified by a factor of 10,000. We recorded from 13 electrodes that were positioned at Fpz, F5, Fz, F6, T7, C3, Cz, C4, T8, P5, Pz, P6, and Oz (10/10 configuration) with the right earlobe as reference. Before recording we confirmed that each electrode’s connection to the scalp had an impedance below 25 kΩ. Data were acquired at a sampling rate of 1000 s^-1^ with a data-acquisition card (PCI 6221, National Instruments, USA) and a program custom-written in MATLAB (MathWorks, USA). The program also provided the subjects with musical signals that were synchronized with the EEG recordings. Music was presented through electrically shielded earphones (hf5, Etymotic, USA) at a comfortable root mean square sound pressure level of 75 dB SPL. The sound intensity was calibrated with a microphone (ECM8000, Behringer, Germany).

### EEG Signal Analysis

Electroencephalography recordings were initially assessed for artifacts such as short, large-amplitude spikes. Recordings with a moderate or large number of artifacts were discarded and the corresponding experiment was repeated. For every channel we then computed the complex Fourier spectrum of the voltage time series. We extracted the amplitude and phase of the Fourier component at the beat frequency (6 Hz). We then selected beat-responsive channels, that is, channels that had an amplitude at the beat frequency that significantly exceeded those at neighboring frequencies. To determine significance, we computed the mean and SD of the amplitudes at frequencies of 5–7 Hz, excluding the beat frequency. If the amplitude at the beat frequency exceeded the mean over the neighboring frequencies by more than two SDs, we denoted the channel as beat-responsive. For all recordings with the exception of one trial, significant responses emerged at the beat frequency. The trial without significant responses was for Subject 6 in response to the stimulus ‘TT’; this trial was discarded. Scalp topographic maps of the EEG voltage amplitude at the beat frequency were produced with the open-source Matlab toolbox EEGLab (Delorme and Makeig, 2004).

We computed the average amplitude and phase at the beat frequency, as well as the corresponding SEM, over all beat-responsive channels. To this end we computed the average time series from beat-responsive channels. We divided each two-minute time series into 120 segments. The Fourier transform of each segment was calculated and the complex Fourier coefficient at the beat frequency extracted. From the complex coefficients of all segments we computed the average amplitude at the beat frequency as well as the SEM. We also conducted a one-sample Kolmogorov–Smirnov test on the complex Fourier coefficients for each of the different segments to confirm that the data originated from standard normal distributions. This procedure validated the use of Student’s *t*-tests for the comparison of EEG responses.

We checked for stimulus artifacts by recording the EEG response when the earphones were activated near the ear but not inserted into the ear canal. A subject could then not perceive the music and the response at the beat frequency was absent from the EEG recording.

### Statistical Analysis

For the first experiment we compared the EEG response to a musical piece with the response to its scrambled version. For the second experiment we compared the EEG response to a musical piece for a subject when attending the music to that during reading of text. In both cases we investigated differences in the EEG amplitudes at the beat frequency, averaged over all beat-responsive channels. We then assessed the statistical significance of the differences on three levels: individual trials, individual subjects, and the population.

At the level of individual trials we investigated whether statistically significant results could be obtained from single trials, which is important for a potential use in brain–computer interfaces and medical diagnostics. We used a paired two-sample Student’s *t*-test to assess whether any difference in the average amplitude from the two EEG responses of a single trial was statistically significant.

The level of individual subjects indicates whether statistically significant differences can be obtained from all trials with a single subject, which is necessary for the use of this method in clinical assessments. For each subject we computed the mean and the SEM of the difference in the response between the two conditions. Through a one-tailed one-sample Student’s *t*-test we then determined the statistical significance of the mean’s exceeding zero for music comprehension or being smaller than zero for attention.

At the population level we explored whether the averages of the response differences across subjects were statistically significant and thus whether there was a consistent population behavior. We computed the mean and SEM of the differences in responses from all subjects. We employed one-tailed, one-sample Student’s *t*-tests to determine whether the averaged differences in the responses were significantly larger than zero for music comprehension or smaller than zero for attention.

### Statistical Classification

To explore a potential use of our recording paradigms for the clinical assessment of auditory processing and music cognition and in brain–machine interfaces, we sought to determine whether we could employ techniques from machine learning to accurately classify the cortical responses.

For classification we considered the EEG amplitude at the beat frequency for the different individual channels as potential features. We investigated the results from single trials as well as from the average over all four trials with a given subject. Because the activity of the cerebral cortex can change over time and baseline values in EEG recordings can accordingly shift, we computed the difference in the amplitudes at each electrode between the paired recordings in each trial. For the experiment on music comprehension we computed the difference between the EEG responses to a scrambled musical piece and those to the original tune. For the experiment on attention we computed the difference in the EEG responses during reading of the text to that during listening to the music. In both cases, these differences constituted one category of data. A second category was obtained by inverting the sign of the obtained differences. The second category thus contained the difference between the EEG response to a musical piece and that to its scrambled version or the difference between the EEG response during listening to the music and that during reading of the text.

We sought to classify both categories through supervised learning (McLachlan, 2004; Lotte et al., 2007). We first reduced the number of features from the original 14 through a genetic algorithm, the forward stepwise-regression model (Mitchell, 1998). This method identifies the most informative features using a greedy selection approach. We then applied linear discriminant analysis (LDA), a statistical method that fits a multivariate normal distribution to each class, with a pooled estimate of covariance, and finds the best linear separator. To find a classifier that was maximally robust to inter-trial variations, the classifier was trained on the data from the individual trials. We used cross-validation with 100 iterations. In each iteration, the data were divided into 10 partitions. The classifier was then successively trained on 9 of the 10 partitions and was tested on the remainder. This classifier was then also tested on the data obtained from averaging over the four trials from each subject.

## Results

We found that EEG responses reliably tracked the beat frequency of a melodic stimulus (**Figure 1B**). The measured response to the beat was largest in the frontal, fronto-parietal, and central scalp areas; smaller in the temporal and parietal areas; and smallest in the occipital area (**Figure 1C**). Over the frontal area, for instance, the EEG response obtained from a two-minute recording was typically more than fivefold as great as the responses at neighboring frequencies. Eleven of the 13 channels most often had responses at the beat frequencies that significantly exceeded those at neighboring frequencies; we denote these as beat-responsive channels. The EEG response to the beat is thus a reliable marker for the cortical response to the musical stimuli.

### Comprehension of Music

We investigated whether the EEG response to the beat can signal the comprehension of music. To this end we created four segments of familiar tunes. For each piece we then produced a scrambled version in which segments of notes were shuﬄed. The envelopes of the scrambled versions had the same frequency spectra as those of the original pieces; in particular, they had the same beat amplitude (**Figure 1B**). Moreover, the scrambled versions had the same pitch changes as the original tunes. The musical tunes and their scrambled counterparts thus agreed in their low-level structure, but differed regarding their high-level content: the scrambled pieces lacked a recognizable tune and were non-sensical. EEG recordings were obtained from four trials during each of which a subject listened to one of the four musical pieces as well as to its scrambled version.

**FIGURE 2:**
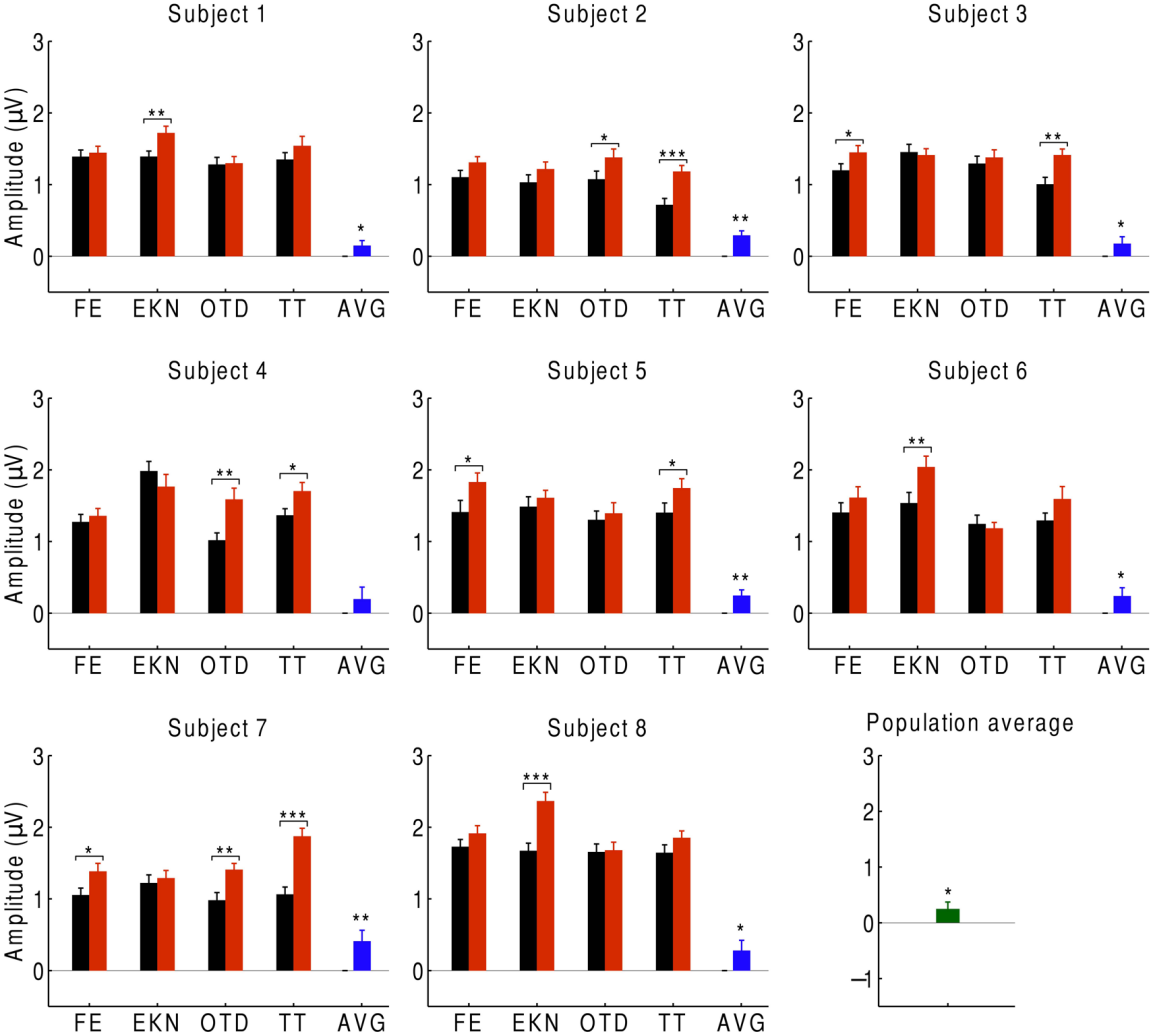
**Cortical responses to musical pieces and their scrambled versions**. For eight subjects, we show the EEG responses at the beat frequency during listening to musical pieces (black) and to their scrambled versions (red). The EEG responses are the averages over the signals of the beat-responsive channels. Each subject experienced four trials with distinct musical pieces, abbreviated as FE, EKN, OTD, and TT. Error bars denote the SEM. The differences in the response were statistically significant in about half of the trials (*p < 0.05; **p < 0.01; ***p < 0.001). The average differences in the EEG responses at the level of individual subjects were significant in all but one subject (blue). The population average—that is, the differences in EEG responses averaged over all subjects—was significantly positive as well (green).

We first investigated the average over the signals at beat-responsive channels. We found that, at the population level, the EEG response at the beat frequency was significantly smaller (*p* < 0.05) during listening to the musical piece than during listening to the scrambled version of the musical piece (**Figure 2**). This result similarly emerged when investigating individual subjects: every subject had a smaller response to the musical tune than to its scrambled version, and the difference was statistically significant (*p* < 0.05) for all subjects except for one who had a difference on the verge of significance (*p* ≈ 0.05). Moreover, statistically significant differences in the neural responses could be obtained from about half of the individual trials.

**FIGURE 3:**
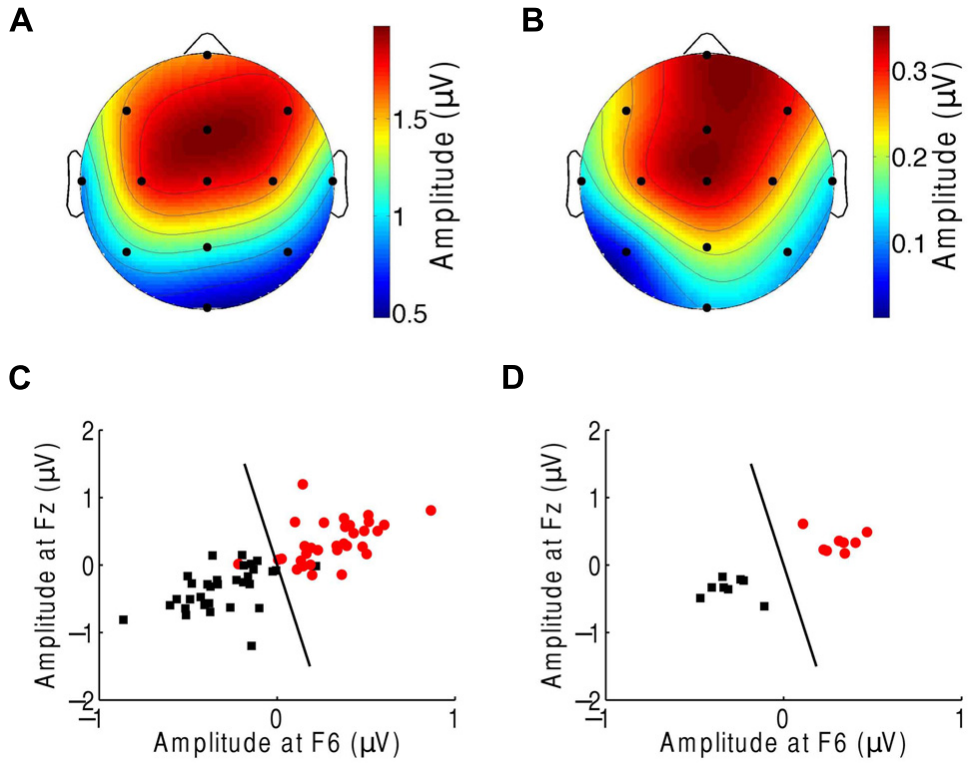
**Classification of the neural responses to musical pieces and their scrambled versions. (A)** The EEG response to scrambled musical tunes, at the beat frequency, was greatest near the frontal and central areas. We show the average of the amplitude over all trials and subjects. **(B)** The frontal and central areas also exhibited the largest difference in EEG amplitude at the beat frequency upon comparison of the response to a musical piece with that to its scrambled version. We show the differences between the cortical response to random musical stimuli and to the original musical tunes, averaged over all trials and all subjects. **(C)** The category of the response difference between a scrambled musical piece and its original version (red circles) could be distinguished from the category of the inverse differences, namely the difference in the response to a musical piece and its random counterpart (black squares), based on the single trials from all subjects. Highly accurate discrimination (black line) of the two categories was achieved by the EEG response at the beat frequency at the frontal channels Fz and F6. **(D)** The classification was completely accurate when we considered the averages over all four trials from a given subject.

We then sought to identify which of the EEG channels or which combination of channels was most informative regarding the comprehension of music. Although the topographic response across the scalp was similar in subjects in response to both the normal and scrambled musical pieces (**Figures 1D and 3A**), and although all scalp regions had on average a stronger response to the beat of scrambled music than to the original tunes, the differences were largest in the central and frontal areas (**Figure 3B**). This result suggests that a few channels suffice to discriminate successfully between the perception of a familiar musical tune and that of an unrecognizable, non-sensical musical piece.

We next attempted to classify the EEG responses according to music comprehension on the basis of only a few EEG channels. We assembled the EEG responses into two categories. The first category was the difference in the EEG amplitude at the beat frequency during listening to a scrambled musical piece and that for its original version. The second category was the inverse difference. Because brain activity and thus EEG recordings can change over time, we employed these differences instead of directly using the EEG responses to the musical stimuli. The effects of non-stationarity largely disappeared when we considered the differences in the brain activity for paired recordings that were obtained successively.

We then applied supervised machine learning to identify which channels could best discriminate the two categories and used those channels for classification. We found that both categories could be reliably differentiated, based on the individual trials from all subjects, with only two features, the EEG responses at channels Fz and F6 (**Figure 3C**). These two features alone correctly classified about 92% of the trials. Moreover, at the level of individual subjects, the classification accuracy was 100% (**Figure 3D**).

### Attention to Music

We also investigated the attentional response to the beat of music. Subjects were presented with both a musical stimulus and a printed excerpt from a novel (Tiitinen et al., 1993). We conducted four trials with the four different musical pieces. During each trial subjects listened to two presentations of the same musical piece. For one trial they attended to the music whereas for the other they ignored the musical stimuli and read the text.

**FIGURE 4:**
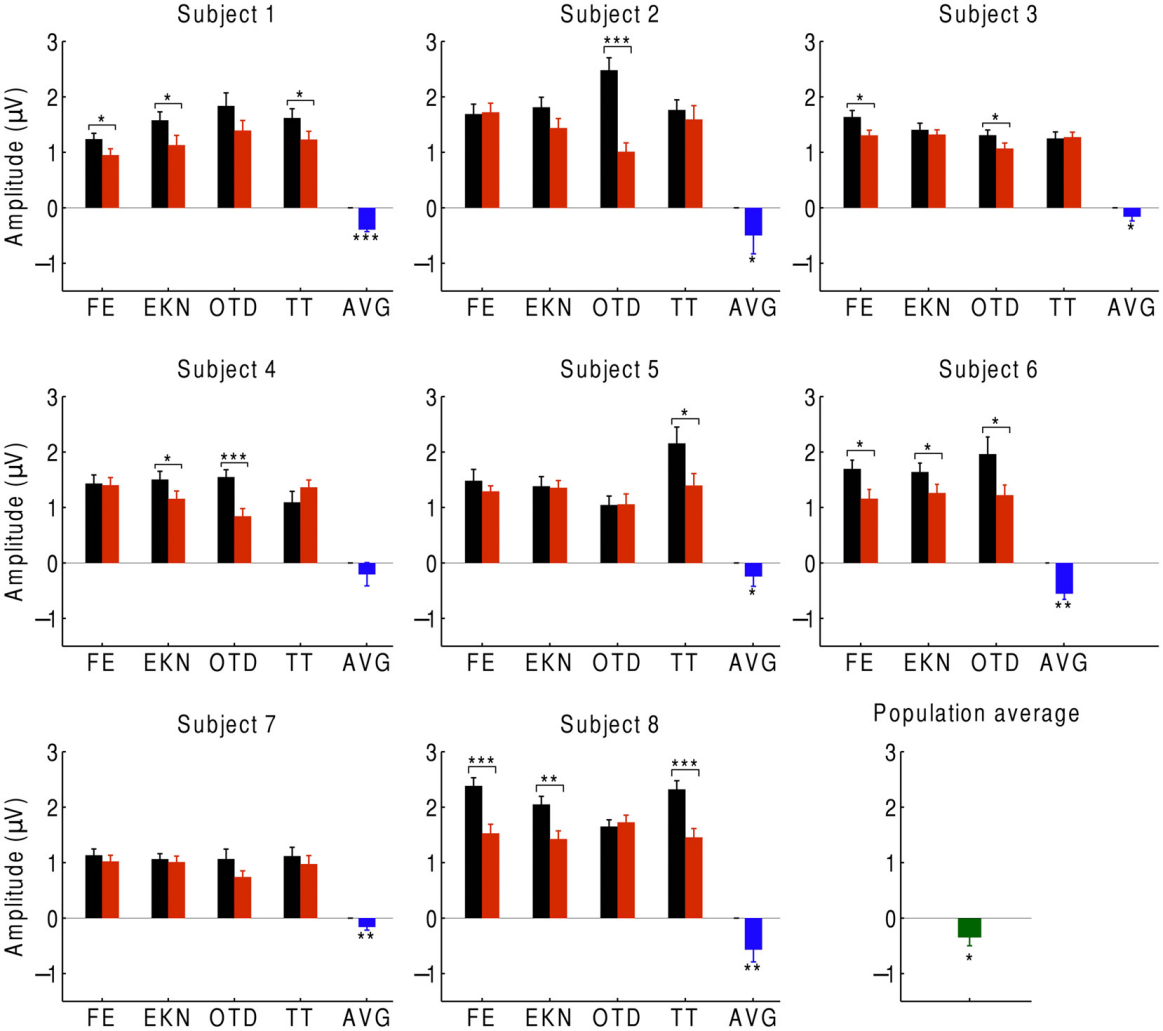
**Cortical responses during attention**. We recorded the EEG responses of eight subjects, averaged over the beat-responsive channels, as they either attended to a musical piece (black) or ignored it (red). The difference in response between ignoring and attending was negative in almost all of the trials, and statistically significant in about half of the trials (*p < 0.05; **p < 0.01; ***p < 0.001). Error bars denote the SEM. At the level of individual subjects, the average difference between the responses in both conditions was always negative and almost always significant (blue). The population average of the response difference was significantly negative as well (green).

In order to investigate auditory attention to the musical input, we averaged the neural responses to the beat over the EEG channels with significant responses to the beat (**Figure 4**). We found that, at the population level, the EEG response at the beat was significantly smaller when ignoring the music than when attending to it. We observed the same behavior for every subject: the average difference in the responses between ignoring and attending the music was negative for every subject and significantly below zero for all but one, whose response approached statistical significance (*p* ≈ 0.05). At the level of individual trials, we likewise found that, for 27 of the 32 trials, the amplitude was smaller when ignoring the music. The differences were statistically significant in about half of the trials.

**FIGURE 5:**
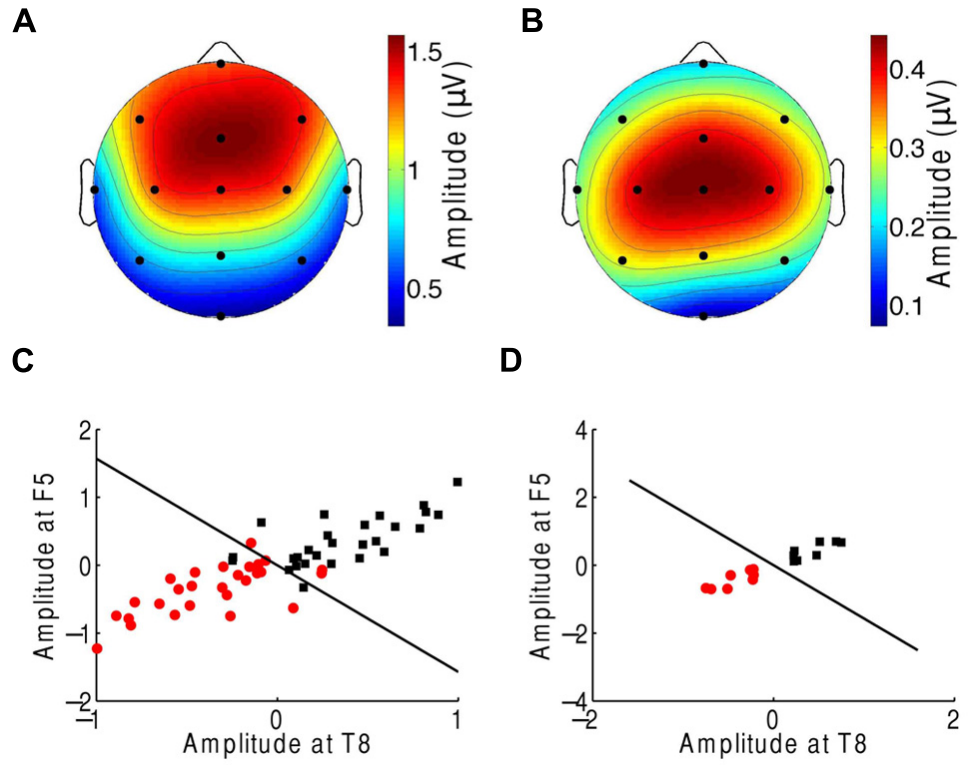
**Neural responses to the beat in music during an attention task. (A)** The frontal and central areas had the strongest response to the beat of music when ignoring the music. We show the average amplitudes over all trials and subjects. **(B)** Averaged across all trials and subjects, the difference in EEG amplitude at the beat frequency for attending to the music vs. ignoring it was largest in the central area. **(C)** The category of differences in the EEG responses between ignoring the music and attending to it (red circles) and the category of the inverse differences (black squares) could be differentiated with only two channels, F5 and T8. The class boundary (black line) was found through linear discriminant analysis (LDA). **(D)** Classification reached full accuracy when we classified the averages over the four trials from each subject, instead of individual trials.

We finally investigated which scalp areas provided the most important information regarding attention. The prefrontal, frontal, and central channels provided the strongest response to the beat of music both during attention to a musical piece (**Figure 1C**) and when ignoring the musical piece (**Figure 5A**). The central, frontal, and temporal regions were particularly informative on attentional modulation of cortical activity (**Figure 5B**). We therefore attempted to classify the EEG responses corresponding to attending to or ignoring the music on the basis of only a subset of EEG channels. As in the experiment on music comprehension, we defined two categories. The first was the difference in the EEG amplitudes at the beat frequency between ignoring the musical stimulus and attending to it. The second category was the inverse signal, that is, the difference in the EEG response at the beat frequency between attending to the music and ignoring it. We found that as few as two channels, F5 and T8, could classify about 88% of all individual trials correctly (**Figure 5C**). Classification was fully accurate when considering the averages over all trials from individual subjects (**Figure 5D**).

## Discussion

Our study shows that there is a reliable neural response to the beat of a melody. Moreover, the experiment demonstrates that the response is weaker for a familiar musical tune than for a scrambled, non-sensical musical piece. The steady-state response to the beat can thus reveal the comprehension of a familiar musical tune. Our finding that a comprehended tune elicits a weaker steady-state response than an unfamiliar one is similar to a previous result on the ASSR evoked by amplitude-modulated speech: the corresponding response in a subject’s left hemisphere was weaker for intelligible than for unintelligible reversed speech (Deng and Srinivasan, 2010). However, the neural response to the beat of music does not show a hemispheric difference (**Figures 1D** and **2**).

Our experiment additionally demonstrates that attention enhances the cortical response to music, which concurs with several previous findings regarding the auditory ASSR (Ross et al., 2004; Bidet-Caulet et al., 2007; Müller et al., 2009). However, unlike the responses in previous studies using amplitude-modulated pure tones, the attentional modulation of the neural steady-state responses to the beat of music that we have described here is not restricted to the left hemisphere but occurs equally in both hemispheres.

The comprehension of music and attention can be inferred not only from the population’s response but also from the responses of individual subjects. Moreover, these cognitive processes can be assessed with high accuracy from the individual trials of a single subject. These assessments can be achieved with only a few selected EEG channels identified through machine-learning techniques. Because music comprehension and attention can be determined reliably from a few minutes of EEG recordings, from a few electrodes, and in a single subject, our paradigms have potential clinical applications. In particular, it will be interesting to assess auditory processing and music cognition in individuals with different forms of amusia and to determine how their cortical responses to familiar and to non-sensical musical tunes differ from the responses of control subjects (Schuppert et al., 2000; Samson et al., 2001; Peretz and Coltheart, 2003). This approach may allow the classification of different forms of amusia and an assessment of specific brain impairments (Musiek and Chermak, 2009).

Our results might also be applied in novel auditory brain–machine interfaces. As an example, paralyzed patients might benefit from an interface activated by attention to music. A patient might answer a binary question by attending or not attending to a musical segment. Furthermore, an auditory brain–machine interface based on the cognition of music could allow the assessment of patients with disorders of consciousness, a task that currently poses major challenges (Giacino et al., 2002; Laureys et al., 2004; Schiff, 2010; Goldfine et al., 2011). Determining the cortical response to the beat of music could inform physicians about a patient’s ability to comprehend music as well as to attend to it, and thus help to assess high-level cognitive functions. Because we have demonstrated that the brain responds to the beat of naturalistically presented music, such an assessment would yield a promising passive screening of brain-injured patients.

An important question remains regarding the origin of the cortical response to the beat. Because we employed tunes with a note at every beat, the beat frequency was prominent both in the stimulus and in its envelope. The cortical response that we measured might accordingly represent a frequency-following response to this feature. Because cortical oscillations exist within the theta frequency band around the beat frequency, the response might alternatively represent the entrainment of these endogenous oscillations to the stimulus. The latter mechanism would resemble cortical oscillations in the alpha range driven by visual stimuli (Kim et al., 2006; Mathewson et al., 2012; Spaak et al., 2014). Further experiments are needed to examine these potential mechanisms in the cortical response to the beat.

The frontal and central EEG channels responded most prominently to the rhythmic musical beats. In the first experiment we found that the right frontal channels can best differentiate responses to familiar musical pieces from their scrambled, non-sensical versions. The corresponding cortical area has previously been found to be involved in the processing of the pulse, beat, and rhythm of music as well as the recognition of music (Schmidt and Trainor, 2001; Tsang et al., 2001; Hallam et al., 2008; Grahn, 2012). In the second experiment we determined that the frontal, temporal, and central channels provide the most information on attention. These results accord with demonstrations that the prefrontal and temporal cortex contributes to auditory attention (Loui et al., 2005; Fritz et al., 2007).

It is instructive to compare the present results with similar studies on speech comprehension and attention to speech. The cortical delta- and theta-band oscillations entrain to the envelope of a speech signal (Horton et al., 2013; Peelle et al., 2013; Ding and Simon, 2014). The entrainment is stronger for intelligible than for unintelligible speech (Peelle et al., 2013; Ding et al., 2014) and is larger for an attended than for an unattended speech signal (Horton et al., 2013). The beat frequency of 6 Hz that we have employed here falls into the theta frequency band of neural oscillations. The cortical response to this rhythmical feature therefore resembles that of a speech envelope. Unlike the entrainment to speech, however, the cortical response to musical beat is smaller for a familiar tune than for a random melody. As with speech, we found that the neural response to the beat was enhanced with attention. It will be interesting to investigate how these similarities and differences in the processing of speech and music arise and what they signify (Zatorre et al., 2002).

The phase of the cortical response at the beat frequency might hold important information as well. We have found that the phase was approximately 3.5 radians and remained constant over the different brain areas. This phase corresponds to a time delay of about 94 ms. The entrainment of neural oscillations to a speech envelope also occurs at a comparable latency of around 100 ms (Ding and Simon, 2012; Horton et al., 2013; Ding et al., 2014). This coincidence again suggests commonalities in the cortical response to and processing of complex auditory stimuli.

Further studies are required to understand how music cognition shapes the response to the beat. For music comprehension in particular we have compared the EEG responses to familiar musical tunes to those of unfamiliar, non-sensical pieces. The observed differences in the cortical responses might originate from the familiarity vs. unfamiliarity of the music, from the different structures of the tunes from Western music vs. their scrambled versions, or from both. Investigating these issues will further our understanding of the fascinating interplay between the structure of music and its neural processing.

## Author Contributions

BM, CR, CB, NS, AH, and TR contributed to the design of the work, the acquisition of the data as well as its analysis and interpretation, drafted and revised the manuscript, approved the manuscript for publication, and agree to be accountable for all aspects of this work.

## Conflict of Interest Statement

The authors declare that the research was conducted in the absence of any commercial or financial relationships that could be construed as a potential conflict of interest.
